# The complete chloroplast genome sequences of a highly Endangered orchid species *Paphiopedilum barbigerum* (Orchidaceae)

**DOI:** 10.1080/23802359.2019.1660269

**Published:** 2019-09-05

**Authors:** Mingyu Li, Zhe Zhao, Jian He, Jin Cheng, Lei Xie

**Affiliations:** a Beijing Laboratory of Urban and Rural Ecological Environment, Beijing Key Laboratory of Ornamental Plants Germplasm Innovation and Molecular Breeding, College of Biological Sciences and Technology, Beijing Forestry University, Beijing, China;; b School of Nature Conservation, Beijing Forestry University, Beijing, China

**Keywords:** *Paphiopedilum*, complete chloroplast genome, Endangered species, Orchidaceae, phylogenomics

## Abstract

*Paphiopedilum barbigerum* (Orchidaceae) is an endangered species with highly ornamental and horticultural value. The chloroplast genome of the species was assembled using next-generation sequencing method. The complete cp genome sequence is 156,329 bp in length, comprising a pair of inverted repeat regions (IRs) of 34,214 bp each, separated by a large single-copy (LSC) region of 86056 bp, and a small single-copy (SSC) region of 1845 bp. The chloroplast genome contains 126 functional genes, including 80 protein-coding genes (PCGs), 38 tRNA genes, and 8 rRNA genes. The phylogenetic position of the species based on the complete cp genome was also inferred in this study.

*Paphiopedilum barbigerum* T. Tang & F. T. Wang native to crevices of shady limestone cliffs, rocks, or tree trunks at an elevation of 800–1500 m in northern and western Guangxi, Guizhou, and southeastern Yunnan Province, China (Liu et al. 2009; Long et al. 2009; Shi et al. 2009). Since species *Paphiopedilum* were popular and considered to be very valuable for Asian gardeners, *P. barbigerum* has been excessively mined (Liang et al. 2014). In addition, the habitat of *P. barbigerum* continuously faced destruction due to deforestation in the past years. By now, this species is very rare and endangered (Liang et al. 2014). It has been listed as an Endangered species (EN) in the Red List of Endangered Species of the World Conservation Union (International Union for Conservation of Nature, IUCN 2015). In order to better understand and protect this species, we reported and characterized the first complete chloroplast genome of *P. barbigerum* by the next-generation sequencing technology. Furthermore, phylogenomic analysis of this species and its relatives was also presented.

Fresh leaf material from *P. barbigerum* was collected from a living individual in the greenhouse of Beijing Forestry University, which was transplanted from Libo County, Guizhou Province (N25.322681°, E107.94811°). Voucher specimens were deposited in the Herbarium of Beijing Forestry University (BJFC) (collection numbers LMY201901). We used CTAB method (Doyle and Doyle 1987) to obtain total genomic DNA. Then, an Illumina HiSeq 4000 platform at Novogene (http://www.novogene.com, China) was applied to perform 2 × 150 bp pair-end sequencing. Clean reads were mapped to publish chloroplast genome of *Paphiopedilum* as references (Kim et al. 2015; Lin et al. 2015; Hou et al. 2018) using Map function of Geneious R11 (Kearse et al. 2012). Filtered reads were then used for *de novo* assembly with Geneious R11. Gaps were bridged using Fine Tuning function of Genious R11. The complete chloroplast sequence was annotated using Plann (Huang and Cronk 2015).

The chloroplast genome of *Paphiopedilum barbigerum* is 156,329 bp in length. It comprises a pair of inverted repeat regions (IRs) of 34,214 bp each, separated by a large single-copy (LSC) region of 86,056 bp, and a very short small single-copy (SSC) region of 1845 bp. The chloroplast genome harbors 126 functional genes, including 80 protein-coding genes (PCGs), 38 tRNA genes, and 8 rRNA genes. Among them, 11 protein-coding (*rps*16, *atp*F, *rop*C1, *ycf*3, *clp*P, *pet*B, *pet*D, *rpl*16, *rpl*2, *ndh*B, *rps*12) and 6 tRNA genes (*trna*K-UUU, *trna*G-GCC, *trn*L-UAA*, trn*V-UAC, *trn*I-GAU, *trn*A-UGC) have introns. The average GC content of the complete plastome is 36.0%.

Complete chloroplast genome sequences of *Paphiopedilum* and related genera available from GenBank were downloaded for phylogenomic analysis. Maximum likelihood and Bayesian inference analyses were conducted for phylogeny reconstruction (Figure 1). The sequence alignment and all the settings of ML and Bayesian analyses were the same with the previous study by Kim et al. (2015). Phylogenetic framework of *Paphiopedilum*, as well as its related genera, were consistent with all the previous studies (Kim et al. 2015; Lin et al. 2015; Hou et al. 2018).

**Figure 1.**
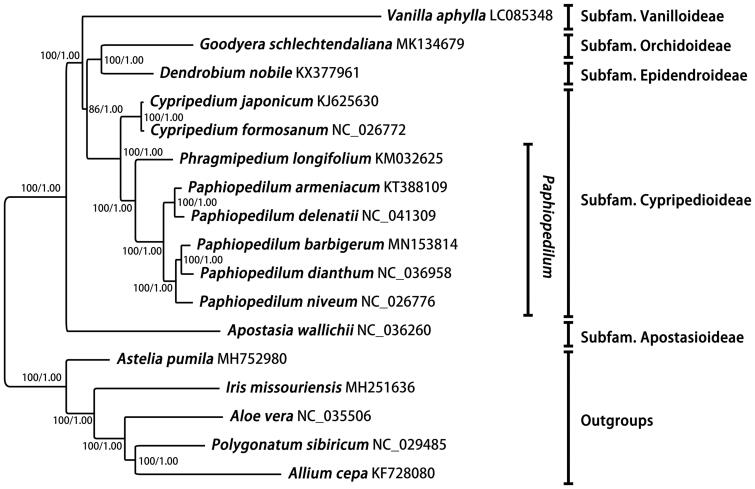
Maximum-likelihood phylogram of *Paphiopedilum* inferred from the complete chloroplast genome sequences. ML bootstrap values/pp values for Bayesian analysis are shown at each node.
